# Soliciting the Oral Route as a Logical Approach to Managing Colon Cancer

**DOI:** 10.3389/fbioe.2021.645923

**Published:** 2021-02-25

**Authors:** Rayan Sabra, Nashiru Billa

**Affiliations:** ^1^School of Pharmacy, University of Nottingham, Semenyih, Malaysia; ^2^College of Pharmacy, QU Health, Qatar University, Doha, Qatar

**Keywords:** colon, cancer, oral, delivery, nanoparticles, formulation, anticancer

## Introduction

According to Global Cancer Incidence, Mortality and Prevalence (GLOBOCAN) 2018, colon cancer (CC) is the third most common cancer in men and second in women worldwide (Bray et al., 2018). Approximately 1.8 million new colon cancer cases were diagnosed globally in 2018, with nearly 881,000 resulting in deaths (Bray et al., 2018). The exact trigger of CC is still subject to debate but it is generally accepted that predisposing factors include genetics, diet and lifestyle. In any case, the chemical basis for cancer initiation cascade appears to be prompted by byproducts of aerobic metabolism such as reactive oxygen species (ROS). These are known to confer various levels of reactivity to biological tissues which may serve as trigger to cancer development (Finkel, 2011). This has been referred to as oxidative stress and is known to cause damage to lipids, proteins and importantly, DNA. On the other hand, ROS can also regulate other biological processes. It would seem therefore that a good balance in the levels of ROS is necessary within biological systems and any imbalance in this level serves as trigger for cancer. Most CCs develop slowly as polyps and eventually become malignant under uncontrolled growth that begins within the colonic mucosa and then spreads to the rest of the colon layers as a solid tumor. Fortunately, CC can be easily treated if detected early. Treatment and prognosis rely on the depth of the tumor, extent of lymph nodes involvement and metastasis to distant parts of the body (Ong and Schofield, 2016). Chemo- or radiotherapy may be used alone or in combination for long term treatment plans, whilst surgery may be required in severe cases. In radiotherapy, X-ray energy is irradiated to the suspected tumor from a linear accelerator that destroys cellular DNA and thus inhibits cell proliferation (Jong, 2017). On the other hand, chemotherapy relies on use of cytotoxic agents that inhibit cell growth (Wu et al., 2012) and may be administered prior to or after surgery. Chemotherapeutic agents commonly used in the management of CC include 5-fluorouracil (5-FU), irinotecan (CPT-11), leucovorin (l-LV), oxaliplatin (L-OHP), and capecitabine (Van Der Jeught et al., 2018). 5-FU was among the first synthesized anti-cancer drugs, however about 85% of parenterally administered 5-FU dose is metabolized within 15 min into inactive forms (Jordan, 2016). Thus, capecitabine, an orally administrated prodrug of 5-FU, is usually used instead due to the higher tumor response rate, ≈100% bioavailability and lower incidence of side effects (Miura et al., 2017).

The majority of chemotherapeutic drugs are administered intravenously (i.v.), destined to accumulate within tumor regions. However, i.v. administration causes significant distribution in highly perfused organs (e.g., kidney, lung, etc.) compared to tumor sites. This untoward deployment of the i.v. administered chemotherapeutics may impair the functionality of said organs upon long-term exposure (Attili-Qadri et al., 2013; Patel, 2014). Thus, research focused on alternative modes of delivery of therapeutics is warranted. In this regard, soliciting the oral route for delivering anticancer agents in the management of CC seems logical because of the manifestation CC within the gastrointestinal tract (Sharma and Saltz, 2000; Date et al., 2016). Oral administration of therapeutics for CC enables localized deployment at tumor and hence improves its efficacy whilst at the same time reducing systemic toxicity. Furthermore, oral delivery route appropriate for the delivery of anticancer agents destined for the treatment of CC because it reduces stress and discomfort to patients, and offers flexibility in that, they can self-administer the medication and thus forgo hospital visits (Eek et al., 2016). Rectal administration presents a possibility for delivering drugs to the colon however therapy is ineffective if CC is widespread or in the event of local inflammation such as in Inflammatory Bowel Disease (IBD) (Hua, 2014). The unidirectional flow of colonic content is bound to void any device inserted rectally, which may also cause discomfort in patients.

Oral administration of drugs destined for therapeutic effects within the GIT increases their availability locally than when administered i.v. This necessitates higher i.v. doses and systemic exposure, which exacerbate side effects akin to the drug (Patel, 2014). A key consideration in the localized delivery of therapeutics to the colon via the oral route is that the delivery system must offer protection to the drug from premature release in the upper gastrointestinal tract. Premature release may render the drug to multi-drug efflux pumps, unfavorable gastric pH, and metabolic enzymes (e.g., cytochrome P450 enzymes) present within the epithelia of the upper GIT (Katragadda et al., 2005). Drug delivery systems able to surmount the aforementioned constraints presented in the upper GIT until arrival at the colon are termed “colon-specific delivery systems.” Five main strategies have been explored by researchers for directing drugs effectively to the colonic region whilst evading aforementioned constraints of the upper GIT (Philip and Philip, 2010). These include (i) strategies based on mediation of ROS levels (ii) pH-dependent, (iii) pro-drug, (iv) time-dependent, and (v) microflora-activated systems. Each of these systems can potentially be employed to deploy anticancer agents to the colon in the management of colon cancer. Some of these systems have also been utilized for treating local inflammatory diseases like IBD or Chron's syndrome, and others are being studied for possible systemic delivery. In the following sections, we will first present the constraints to delivering drugs orally to the colon in colon cancer management and then subsequently, review the evolution of these approaches in colon targeted delivery of anti CC agents via oral administration.

### Constraints to Delivering Drugs to the Colon

The gastrointestinal tract is a tube commencing from the mouth and ending in the anus. It presents formidable physical, physiological anatomical barriers to orally administered drugs destined for therapeutic action at colon. The variable pH along the gastrointestinal tract requires that the dosage form provides necessary protection until deployment at the colon. Following oral administration, the dosage form transits in the stomach prior to emptying in small intestine. With the acidic pH of ~1.2, acid-labile drugs are destined to degradation if the transit in the stomach is prolonged. On the other hand, drugs that are unstable in alkaline milieu will suffer degradation in the small intestine in the absence of formulation interventions. Furthermore, the hydrodynamics within the gastrointestinal tract may serve as a trigger for premature drug release from dosage forms. The situation is compounded by the food status, which not only affect the hydrodynamics of the gastrointestinal track, but may also interact with the dosage form or drug in diverse ways. The ultimate rate and extent of release of the drug from the dosage form at the colon depends on all the factors highlighted above (Billa et al., 2000). It is necessary that the delivery system retains a significant amount of the drug payload at the colon to have meaningful therapeutic impact. Depending on the type of delivery system, the release of the anticancer agent at the colon may prompt therapeutic action. For submicron delivery systems, it may be desirable for the delivery system to be phagocytosed along with the drug cargo by the cancer tissue. Thus, formulators with interests in oral delivery of anticancer agents in colon cancer must be cognizant of these constraints in order to achieve best therapeutic outcomes.

### Orally Administered Chemotherapeutics Based Mediation of ROS Levels in Treating CC

In section Introduction, we highlighted the role of ROS in triggering the cascade of events leading to the development of cancer. However, drug-induced apoptosis through modulation of cellular levels of ROS can be used as an effective anticancer strategy. Crucially, evidence points to significant increase in cellular ROS levels, mediated by some chemotherapeutic agents as the hallmark to manifesting anti CC activity (Sreevalsan and Safe, 2013). Ascorbic acid, emodin, quercetin, curcumin, and host of others anticancer agents are now known to act by ROS mediation (Gandhy et al., 2012). Evidence to this mediation is partly borne by the attenuation of anti CC effects by antioxidants. Curcumin is of particular interest in orally administered chemotherapeutics for CC because of its poorly systemic bioavailability following oral administration. This limitation has been exploited by several researchers for local deployment to the colon (Wong et al., 2019). This aptly, has prompted an explosion of investigations in the development of anti CC formulations of curcumin destined for colonic delivery, most based on novel technologies (Wong et al., 2019). It is likely that the scope of drugs with ROS anti CC mediation will grow among orally administered chemotherapeutics.

### Orally Administered Prodrug-Based Approach in Treating CC

Some of the earlier strategies in delivering drugs orally to the colon were based on prodrugs, whereby the physicochemical properties of the drug were altered through conjugation with hydrophilic molecules (e.g., amino acids and sugars) or polymers (e.g., poly (L-aspartic acid) and dextran) (Jain and Jain, 2008). Hydrophilicity restricts absorption of the prodrug in the upper GIT until colon arrival where, enzymatic degradation by azo-reductase and glycosidase produced by the microflora degrade the prodrug to release active drug. Prodrug formulation has also been exploited in the development of diverse anti-cancer drugs. One such example is capecitabine, the oral prodrug of 5′-deoxy-5-fluorouridine (5′-DFUR) that can be converted to the active drug, 5-fluorouracil (5-FU), by enzymatic degradation for the treatment of breast, colorectal, and gastric cancer. Capecitabine is usually absorbed through the intestine and its conversion occurs in both tumor and normal tissues; nevertheless, the enzyme is detected at higher concentrations in tumors, enabling distinct activation of the drug and for low systemic toxicity in normal tissue (Koukourakis et al., 2008). In a study by Twelves et al., the efficacy of capecitabine vs. intravenously administered 5-FU as adjuvant therapy for stage III CC showed that oral capecitabine is an effective contender to the latter with efficacy benefits retained for 5 years (Twelves et al., 2012). In another study Mohammed et al., the anti-folate chemotherapeutic, methotrexate (MTX) was transformed to MTX-imidazole and loaded in chitosan (CS), to form CS-MTX conjugate as colon cancer prodrug. They showed that the synthesized conjugate is stable at acidic conditions and its anti-proliferative effects were boosted in a dose-dependent manner compared to free MTX. Thus, it was concluded that CS-MTX is a probable human colon cancer prodrug (Mohammed et al., 2020).

### Orally Administered Time-Dependent Systems in Treating CC

Time-dependent systems are designed to release their payload in the colon at a pre-set lag time following oral administration, mainly being controlled by the coating layer used in the system (Gazzaniga et al., 2006). A typical example is the Time Clock® system coated with hydrophobic polyoxyethylene sorbitan monooleate and water soluble hypromellose in which the coating disintegrates in an aqueous environment at a rate proportional to the coating thickness and gradually exposes the drug core for dissolution (Pozzi et al., 1994). Pulsincap®, was the first formulation developed based on this system, whereby its main body is covered with an enteric polymer that dissolves in the small intestine and the concentration of the hydrogel plug specifies the time at which the content are released (Jain and Chourasia, 2003). In a study by Patel et al., a modified pulsincap dosage form of 5-FU was developed to target colorectal carcinoma. The capsule body was made water insoluble by exposing the body to formaldehyde vapor and the *in vitro* drug release was studied at pH 1.2 for 2 h; pH 6.8 for 3 h, and pH 7.4 for 12 h. The study revealed that the modified pulsincap delivery of 5-FU was effective in providing controlled zero-order release after a 5 h lag time (Patel et al., 2011). Nevertheless, time-dependent systems are unreliable due to wide variations in gastrointestinal transit times, influenced by geometry of the delivery system, food intake, inter-subject differences in gastrointestinal motility patterns (Philip and Philip, 2010).

### Orally Administered pH-Dependent Systems in Treating CC

Orally administered pH-dependent devices employ pH changes along the gastrointestinal tract to control drug release. A wide range of coatings made from pH-responsive polymers, such as Eudragit® L100-55, Eudragit® S100 and hydroxypropyl methyl cellulose phthalate have been studied extensively for colon-specific delivery (Jain and Jain, 2008). These polymers have high pH thresholds (at least 5.0) for dissolution and are thus able to resist disintegration in the gastric acidic and small intestinal fluids for several hours. However, colonic specificity of pH responsive dosage forms is questionable due to inter-subject variability in resting gastrointestinal pH values and the fact that anatomical are defined but depend on other factors such as food status and some health conditions. This imprecision in gastrointestinal pH may cause premature drug release (Yang et al., 2002; Ibekwe et al., 2008). In a study by Asfour and Mohsen, the flavonoid, rutin, was loaded to pH sensitive nanospheres, using Eudragit S100® (methyl polymethacrylate), to develop an oral formulation that targets rutin, in a more solubilized form, to the colon (Asfour and Mohsen, 2018). A negligible (<3.5%) rutin release was observed for up to 2 h at pH 1.2. Moreover, <10% of rutin was released from the nanospheres at pH 6.8 for up to 5 h. On the other hand, a substantial amount of rutin was released, from the nanospheres colonic pH value (pH 7.4) since the methacrylate moieties dissolve rapidly upon de-protonation of carboxylic acid groups at pH > 7. In another study, Mishra et al., loaded 5-FU into pH responsive hydrogels for anti-tumor activity against CC cells. They showed that the hydrogel exhibited burst effect up to 93.2% in pH 7.4 buffer after 24 h, concluding that the permeability and release rate of 5-FU were largely affected by the pH of the medium and amount of water in the hydrogel (Mishra et al., 2014).

### Orally Administered Microflora-Activated Systems in Treating CC

Microflora-activated systems typically refer to colon-specific carriers based on polysaccharides which can be digested by bacteria available at the colon. The colonic bacteria usually produce a number of enzymes such as: glucoronidase, xylosidase, arabinosidase, galactosidase, etc. that act on undigested substrates from the small intestine (Sinha and Kumria, 2003). Substrates typically include natural polysaccharides such as chitosan, alginate, guar gum, dextran, and pectin which can also be used as coatings in delivery systems (Jain and Jain, 2008). The vast majority of research on these systems have been dedicated to natural polysaccharides due to their availability, affordability, flexibility, and biocompatibility (Philip and Philip, 2010). These polysaccharides shield the drug from the environments of stomach and small intestine and are able to deliver the drug to the colon. On reaching the colon, they undergo degradation by enzyme or break down of their polymer back bone leading to reduction in their molecular weight and thereby loss of structural integrity. Subsequently they are unable to hold the drug entity any longer, which prompts drug release. Because the biodegradable enzymes are present only in the colon, the use of biodegradable polymers for colon-specific drug delivery seems to be the more rational site-specific approach, when compared to the other approaches. However, like the other systems, limitations to the implementation of this system are premature drug release in the upper GIT due to the swelling of the carrier and inter-subject variability in microbial variety and populations (Rubinstein, 2005). In a study by Rai et al., 5-FU was loaded into dextran microspheres coated with Eudragit® S100 for colon targeting. Their findings demonstrated that dextran protected the microspheres from breakdown in the upper gastrointestinal tract so that the 5-FU load was predominantly released in the colon. Moreover, their organ distribution study suggested that the release of 5-FU from the microspheres in colon was due to the degradation of dextran by the colonic enzymes (Rai et al., 2016). Similarly, Paharia et al. studied the release of 5-FU from Eudragit-coated pectin microspheres in simulated colonic medium containing rat caecal contents under anaerobic conditions where, 70–80% of 5-FU was released within 8 h (Paharia et al., 2007). In another study, chitosan-pectinate nanoparticle was used to orally deliver curcumin, an anti-cancer polyphenol, for the treatment of CC (Alkhader et al., 2018; Sabra et al., 2019). In these studies, the carrier provided protection to curcumin from acidic degradation with a null release of curcumin in the upper gastrointestinal tract. Besides, a significant release of curcumin was observed at media representing the colon. They concluded that the chitosan-pectinate conjugate may serve as a suitable delivery system for curcumin to the colon in which the integrity of chitosan-pectinate matrix is triggered by colonic enzymatic effects.

## Future Prospects

From the forgoing, we note that the oral route remains as the most rational means for administering anti-colon cancer agents. On the other hand, the gastrointestinal tract presents challenging anatomical features, physiology and hydrodynamics that constrain the free delivery of therapeutics to the colon. Research aimed at addressing these constraints are evolving into intelligent systems, some of which depend on the same innate physiology that impede normal delivery of anticancer agents administered orally. Nanoparticulate dosage forms have the added advantage of possible uptake by colon cancer tissue due to their dimension. The use of antibodies (e.g., monoclonal) imparts selectivity to the delivery system for cancer tissue and reduces sporadic therapeutic effects of the anticancer agent on normal tissue. Pharmacogenomics backed by combinatorial anti-colon cancer therapeutics has immerged as the prospective frontier in the management of colon cancer. Along with the formulation of intelligent delivery systems administrable orally, we will be traversing a significant threshold in the treatment of colon cancer.

## Author Contributions

All authors listed have made a substantial, direct and intellectual contribution to the work, and approved it for publication.

## Conflict of Interest

The authors declare that the research was conducted in the absence of any commercial or financial relationships that could be construed as a potential conflict of interest.
